# Optimised laser microdissection of the human ocular surface epithelial regions for microarray studies

**DOI:** 10.1186/1471-2415-13-62

**Published:** 2013-10-26

**Authors:** Bina B Kulkarni, Desmond G Powe, Andrew Hopkinson, Harminder S Dua

**Affiliations:** 1 Division of Ophthalmology and Visual Sciences, B-Floor, Eye & ENT Building, Queen’s Medical Centre, Derby Road, Nottingham, UK; 2 Department of Pathology, A-Floor, West Block, Queen’s Medical Centre, Derby Road, Nottingham, UK

**Keywords:** Ocular surface epithelium, PALM laser microdissection, Limbal epithelial crypt, Limbus, Cornea, Spotted oligonucleotide microarrays, Gene 1.0 ST array

## Abstract

**Background:**

The most important challenge of performing *insitu* transcriptional profiling of the human ocular surface epithelial regions is obtaining samples in sufficient amounts, without contamination from adjacent tissue, as the region of interest is microscopic and closely apposed to other tissues regions. We have effectively collected ocular surface (OS) epithelial tissue samples from the Limbal Epithelial Crypt (LEC), limbus, cornea and conjunctiva of post-mortem cadaver eyes with laser microdissection (LMD) technique for gene expression studies with spotted oligonucleotide microarrays and Gene 1.0 ST arrays.

**Methods:**

Human donor eyes (4 pairs for spotted oligonucleotide microarrays, 3 pairs for Gene 1.0 ST arrays) consented for research were included in this study with due ethical approval of the Nottingham Research Ethics Committee. Eye retrieval was performed within 36 hours of post-mortem period. The dissected corneoscleral buttons were immersed in OCT media and frozen in liquid nitrogen and stored at −80°C till further use. Microscopic tissue sections of interest were taken on PALM slides and stained with Toluidine Blue for laser microdissection with PALM microbeam systems. Optimisation of the laser microdissection technique was crucial for efficient and cost effective sample collection.

**Results:**

The starting concentration of RNA as stipulated by the protocol of microarray platforms was taken as the cut-off concentration of RNA samples in our studies. The area of LMD tissue processed for spotted oligonucleotide microarray study ranged from 86,253 μm^2^ in LEC to 392,887 μm^2^ in LEC stroma. The RNA concentration of the LMD samples ranged from 22 to 92 pg/μl. The recommended starting concentration of the RNA samples used for Gene 1.0 ST arrays was 6 ng/5 μl. To achieve the desired RNA concentration the area of ocular surface epithelial tissue sample processed for the Gene 1.0 ST array experiments was approximately 100,0000 μm^2^ to 130,0000 μm^2^. RNA concentration of these samples ranged from 10.88 ng/12 μl to 25.8 ng/12 μl, with the RNA integrity numbers (RIN) for these samples from 3.3 to 7.9. RNA samples with RIN values below 2, that had failed to amplify satisfactorily were discarded.

**Conclusions:**

The optimised protocol for sample collection and laser microdissection improved the RNA yield of the *insitu* ocular surface epithelial regions for effective microarray studies on spotted oligonucleotide and affymetrix platforms.

## Background

Human donor eyes are important source of tissue for research into pathogenesis, treatment and prevention of disease [1,2]. Obtaining samples of pure cell population is crucial for transcriptomics studies, as contamination with other adjacent cell populations leads to increased biological noise in the microarray data, which interferes with the detection signals from genes of interest in the tissue samples [3]. Laser microdissection (LMD) allows precise collection of cell populations of interest from heterogeneous tissue and preserves the purity and integrity of RNA samples. This is crucial for the accuracy of the microarray results to determine significant differences in gene expression between treatment and control groups [4].

An oligonucleotide microarray was successfully performed on laser microdissected tissue samples by Ohyama H *et al.* in 2000 [5]. A study on laser microdissected breast tissue samples by Cowherd *et al.* in 2004 showed that the technical variability introduced by amplification and hybridisation experiments is smaller than the actual biological variation for different types of breast cancer tissues demonstrated with differential gene expression in these samples [6]. Samples for transcriptional profiling studies on epidermal basal cells, endometriosis tissue, and osteocytes were successfully collected with “Positioning and Ablation in Laser Microdissection” (PALM®) microbeam systems (PALM Microlaser Technologies GmbH) [7]. This technique grew in popularity as it allowed precise dissection of tissues under direct visualisation by a non-contact technique.

### Principle of LMD technology

**Figure 1:**
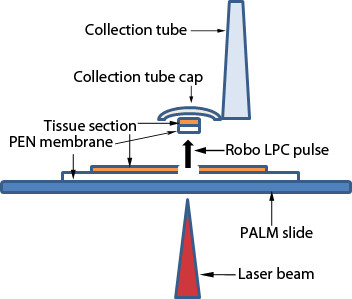
**Schematic overview of LMD process.** Sketch depicts the cap of the collection tube overlying the tissue of interest (orange) mounted on PEN membrane (transparent) coated over the glass slide (blue). Laser beam (red arrowhead) cuts through the PEN membrane and the tissue section. With RoboLPC function, the pulse of laser shot (outlined red arrow) hits the tissue section; and lifts the tissue from the slide surface. The microdissected tissue section is ejected into the collection cap along with the attached PEN membrane.

PALM® microbeam system was used for collection of samples by the non-contact method of pressure catapulting in this study. This system uses a class 1 M UV-A cutting pulsed nitrogen laser (337 nm wavelength) coupled to an inverting microscope, which is focused as 1 μm spot diameter laser beam via the objective lens on the tissue specimen fixed on a PALM® slide coated with polyethylene-naphthalate (PEN) membrane. The PEN membrane facilitates LMD and separation of biological materials such as histological sections, cytospins, cell smears and cell cultures from the slide surface with preservation of the collected tissue morphology [8]. The advantage of this membrane is that it does not interfere with molecular procedures including nucleic acid extraction and amplification. However, the disadvantage of PEN membrane slide is that it cannot be covered with a cover slip therefore, the tissue morphology is difficult to visualise compared to the embedded sections. The laser beam fuses the PEN membrane to the tissue during ablation leading to effective catapulting of the tissue into the collection tube cap with the RoboLPC laser function as the membrane and the tissue acts as a single unit. Laser cutting involves rapid photodecomposition of the tissue in focus; however, the out of focus surrounding tissue is unaffected by heat [9,10]. This unfocussed laser light gets scattered in the surrounding tissue without transfer of the heat energy to the nucleic acids and proteins of the tissue of interest and protects the tissue from the laser burns [11]. This feature maintains the tissue viability of the collected samples [12-14]. Laser catapulting prevents contamination of the tissue by avoiding physical contact with the specimen (Figure 1). Similarly, LMD of other ocular tissues could be performed following appropriate sample preparation. Prior to beginning complex downstream molecular experiments on the LMD samples it is crucial to perform a pilot study for optimisation of the sample processing and LMD with the aim to consistently achieve RNA samples of adequate quality and quantity for successful downstream analysis and to evaluate feasibility, reliability and cost effectiveness of the procedure. Determining the RNA quality and quantity of the samples would help with planning of the experimental design including appropriate selection of suitable microarray platform and the number of biological replicates required for the study.

This article describes in details, the methodology of obtaining laser microdissected RNA samples from the human OS epithelial regions such as the cornea, limbus, conjunctiva and Limbal Epithelial Crypt (LEC) for transcriptional profiling with consideration of factors influencing the quantity and quality of these samples such as staining procedure, duration of LMD and the area of tissue dissected.

## Methods

### Materials and supplies

15 mm trephine

2% v/v povidone-iodine solution

Optimum temperature compound (OCT, Emitech Ltd, East Sussex, England)

Absolute ethanol

Jung CM 1900 cryostat (Leica Microsystems Ltd., Milton Keynes, UK)

Isopentane

PALM® collection tube (PALM, Bernried, Germany; Product code 1440–1000)

70% v/v ethanol

RNase free 0.1% w/v Toluidine Blue

0.1% diethyl pyrocarbonate (DEPC) treated water

1% β-Mercaptoethanol (Sigma Aldrich, Gmbh, Germany)

RNeasy Micro kit (Qiagen, West Sussex, UK)

PALM Membrane slides (PALM # 1440–1000)

LPC Microfuge tubes, 2 mm rim, 500 μl (PALM # 1440–0200)

Latex disposable gloves

PBS

11 mm scalpel blade

Cryospray (Cell Path Ltd, UK)

Ambion® Message Amp™ II aRNA Amplification Kit

NuGen WT-Ovation™ Pico RNA Amplification System

### Ethical approval and retrieval of the cadaver eyes

This research project was approved by the Nottingham Research Ethics Committee and the Research and Development department of the Nottingham University Hospitals National Health Service Trust. Donor eyes not used for transplantation but also consented for research by the relatives of the deceased, and eyes harvested for research purpose only were included in the study. Retrieval of the cadaver eyes was performed with conventional techniques within 24 to 36 hours of death to maintain the cellular RNA viability of the OS epithelium. Processing of the tissues following prolonged post mortem interval could affect the quality of the samples due to degradation of tissue RNA and drying of the OS epithelium following atmospheric exposure. Quality of the OS epithelium in the donor eyes was assessed with the dissecting microscope prior to the tissue preservation.

The inclusion criteria for donor eyes was: i) Donor age between 20 to 70 years; ii) Donors of either sex; and iii) Eyes with healthy OS epithelium. Donor eyes selected for research were logged in an eye tissue database for use in the study.

### Ocular tissue processing and preservation

To prevent RNase contamination of the donor tissue the following precautions were taken; cleaning bench tops and lab equipment with Trigene and Ethanol (EtOH), use of disposable gloves, sterile disposable instruments, petridishes and blades. Whole eyeball supported on an eye stand was cleaned by immersing in 2% v/v povidone-iodine solution for two mins, followed by a sterile PBS wash. A corneoscleral button with 3 mm frill of conjunctiva surrounding the limbus, to include palisades of Vogt and LEC was dissected from the cadaver eye with a 15 mm trephine [15]. The corneoscleral button was placed in a petridish with PBS to prevent drying of the OS epithelium and cut radially into eight triangular segments with a disposable scalpel blade. Handmade aluminum foil cups of approximately 20 mm diameter were filled with an embedding medium optimum temperature compound (OCT, Emitech Ltd, East Sussex, England). Each triangular segment of corneoscleral button was oriented in the OCT compound, in such a way that one of the long edges of the triangle was parallel to the surface of the OCT medium and short edge perpendicular to the superficial edge. Frozen tissue blocks were prepared by immersing the OCT embedded tissue in Isopentane bath pre-chilled (frozen) in liquid nitrogen. Frozen blocks were stored at −80°C till further use.

### Cryosectioning

Cryosectioning was performed with the Jung CM 1900 cryostat (Leica Microsystems Ltd., Milton Keynes, UK). Collection and processing of the tissue including RNA extraction was performed under RNase free conditions. Thorough cleaning of the cryostat chamber was performed with absolute EtOH and acetone prior to use for prevention of tissue contamination. The tissue sections of interest were taken on PALM slides and fixed for 5 mins in 70% EtOH pre-chilled in the cryostat. The slides were then air-dried briskly and placed in a pre-chilled slide box for storage at −80°C for LMD.

### Pre laser microdissection processing of tissue sections

Immediately, prior to LMD, cryosections on the PALM slides were thawed on ice and stained with RNase free 0.1% w/v Toluidine Blue dye for 30 seconds, by pipetting approximately 200 μl of the dye over each of the tissue section. The stained slides were then rinsed twice in 0.1% diethyl pyrocarbonate (DEPC) treated water and air dried for 5 mins.

### Laser microdissection (LMD)

**Figure 2:**
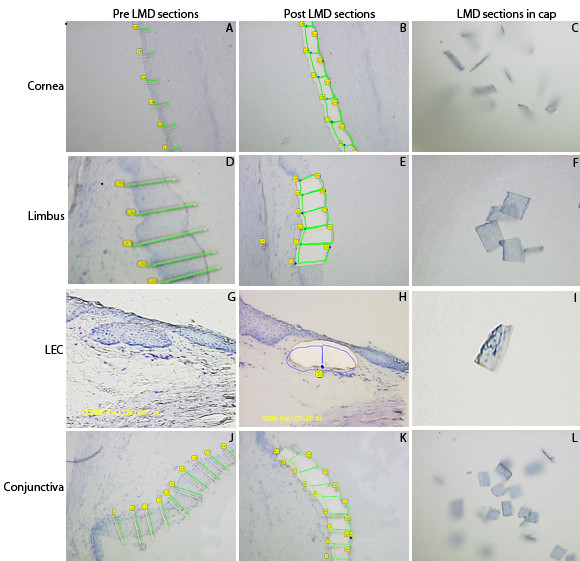
**Laser Microdissection of OS epithelial regions.** The histological OS epithelial sections used for LMD were stained with toluidine blue dye. All the images in the composite are at 20x magnification. Composite shows tissue sections in different stages of LMD. Images **A**, **D** and **J** represents preLMD corneal, limbal and conjunctival epithelium with horizontal graphic lines outline dividing the epithelial width into segments. Images **B**, **E**, **H** and **K** represent post LMD sections of corneal, limbal, LEC and conjunctival epithelium. The graphic outline around the segments is joined to facilitate cutting with laser and the cut segments were catapulted in the collection tubes (images **C**, **F**, **I** and **L**). Image **G** represent preLMD section of LEC, with RoboLPC function the LEC was dissected and catapulted in the collection tube (Image **H**). In image H LEC tissue was sectioned into half to facilitate catapultion in the collection tube. The dot seen at the end of the midsection cut marks the point where the laser pulse catapults the tissue in the collection tube by RoboLPC function (Image **I**).

LMD of the OS epithelium was performed with the PALM® Micro beam system (Zeiss Instruments, Bernreid, Germany). The toluidine stained slide was placed on the automated computer controlled robostage of the laser dissection microscope. The area of interest in the tissue section was brought into focus at 10X magnification within 1 μm precision by the computer controlled movement of the Robostage. Following which, the area of interest in the tissue section was brought to focus at 20X magnification on the computer monitor connected to the microscope and delineated using a computer graphic tool (Figure 2). The cap of a sterile PALM® collection tube (PALM, Bernried, Germany; Product code 1440–1000) attached to the motorised PALM® cap mover was then over hung over the area of tissue section under focus.

The laser parameters such as the focal diameter of the beam, magnification and numerical aperture of the applied objective lens were adjusted for efficient results. The length of the OS epithelial region was divided perpendicularly into smaller segments with the cut function of the laser (Figure 2A, D and J). The cuts were extended lengthwise for complete dissection of the area of interest. Following this, the whole tissue fragment was catapulted in the cap of the collection tube with RoboLPC function, which is a combination of LMD and pressure catapulting (Figure 2B, E, H and K). In the collection tube the tissue segment sticks to the thermostable membrane at the base of the cap. In case a segment failed to eject into the collection tube, the misdirected piece of tissue was catapulted into the collection tube with the auto Laser Pressure Catapulting function (LPC). On completion of LMD, the collection tube was moved directly over the objective lens using the joystick to check for the catapulted specimens in the collection cap (Figure 2C, F, I and L). Thereafter the collection tube was removed and 300 μl of RNA lysis buffer (RLT) (RNeasy Micro kit, Qiagen) containing 1% β-Mercaptoethanol (Sigma Aldrich, Gmbh, Germany) was added to the tube, the collection tube cap was closed, and the tube sealed with parafilm to prevent contamination during storage. The tube was inverted, briefly vortexed and incubated at room temperature (RT) for 20 mins, to lyse the tissue cells sticking to the roof of the cap and release the RNA. Subsequently the RLT tubes were stored at −80°C until further use.

### Histological identification of OS epithelium

**Figure 3:**
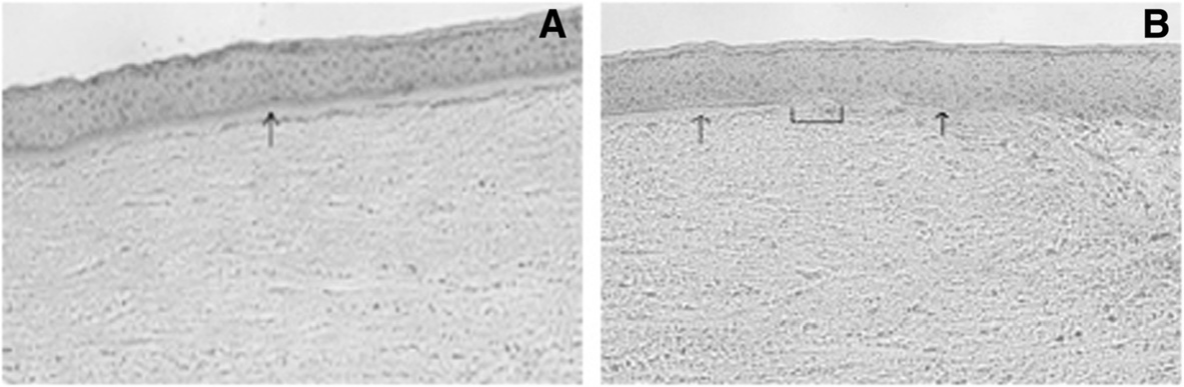
**Composite images of the OS epithelium showing morphology and demarcation zone of corneal and limbal epithelium.** The photograph of the histological cross section of the corneoscleral tissue section stained with haematoxylin and eosin highlights the corneal, limbal epithelium and the underlying stroma. The photograph was taken at 20X magnification. The corneal epithelium is 6 to 8 cell layers thick and is supported by an acellular Bowman’s membrane, which is a double layered refractile structure (marked with a black arrow) (image 3**A**). The termination of the Bowman’s layer also marks the termination of corneal epithelium and the beginning of limbal epithelium. Due to its distinct appearance the Bowman’s membrane is a landmark for identification of corneal epithelium from the adjacent limbal epithelium. Image (3**B**) demonstrates the continuation of corneal epithelium (thin arrow) into the limbal epithelium (thick arrow). The junction of these two epithelia (marked with a drop down box) is an indistinct zone the corneal end of which is demarcated with termination of the Bowman’s membrane and the limbal end of the junction is identified by the beginning of the wavy limbal basal epithelium.

To perform precise dissection of the OS epithelial regions it is crucial to identify the histological landmarks demarcating one zone from the other. Corneal epithelium is characterised by flat basal layer supported by refractile Bowman’s membrane (Figure 3A). The end of the Bowman’s membrane marks the termination of the corneal epithelium and beginning of the limbal epithelium (Figure 3B). The limbal epithelium appears wavy with convolutions due to the loss of support to the basal epithelium. The limbal epithelium is thicker than the corneal epithelium and consists of 10 to 12 layers. The limbal basal epithelial cells are smaller and round with increased nucleus to cytoplasm ratio as compared to the corneal basal epithelium (Figure 3A and B). Microscopic examination of limbal stroma reveals the presence of blood vessels in this region, compared to the avascular corneal stroma. The LEC is a solid cord of cells that extends either parallel or perpendicular from the peripheral aspect of the under surface of limbal palisade of Vogt, into the limbal stroma. LECs vary in size, shape and location around the limbus. Depending on their sizes LECs have been categorised as minor (<40 μm) or major (>40 μm). The junction between limbus and the conjunctiva is marked as a constriction in the OS epithelium, due to change in the limbal epithelial thickness from 10–12 cells to a thickness of 2–5 cells of conjunctival epithelium. The epithelial surface of the conjunctiva is irregular and ragged. Presence of goblet cells in the epithelium is also a feature for identification and confirmation of the conjunctival tissue. Compared to the limbal stroma the conjunctival stroma has dense infiltration of cells along with the presence of accessory lacrimal glands in this region. For precise LMD it is preferable to avoid junctional areas between two regions.

### RNA extraction

Total RNA extraction of LMD tissue was performed with RNeasy Micro kit, according to the manufacturer’s protocols (Qiagen, West Sussex, UK). Each sample was made up to 350 μl with RLT buffer (activated with 10 μl of β-Mercaptoethanol per 1 ml of buffer RLT).

## Results

This section mainly presents the effect of optimisation of LMD technique with consideration to cryopreservation of the tissue, thickness of the cryosections taken on PALM slides, fixation technique and tissue staining.

### Optimisation of sample processing for LMD

Optimisation of the sample preparation and LMD technique was crucial for efficient time and cost management of the project. Practically all the steps of the LMD were optimised, and their effectiveness observed. The findings are described below.

### Freezing of tissue blocks

The tissue processing for LMD was performed taking care to preserve the RNA integrity of the processed tissue. On histological examination, it was noted that tissue sections of tissue blocks snap frozen in liquid Nitrogen developed cracks or freeze fractures. This compromised the tissue quality due to which the tissue had to be discarded. To avoid this problem controlled gradual freezing of the tissue was performed in Isopentane pre-chilled in liquid Nitrogen.

### Thickness of the cryosections

Following optimisation of tissue thickness, cryosections of 6 μm thickness were found to be effective for LMD. It was noted that thin sections (< 6 μm) folded easily or tore while transferring on to the slides. This made the OS epithelium inaccessible to LMD and the affected tissue sections had to be discarded. On the other hand, thick sections (> 6 μm) were easily lost and fell off the slide during fixation and washes. It was also noted that during LMD the thick sections did not catapult effectively into the collection cap and got lost or misdirected over the surrounding tissue.

Consistent fixation of tissue sections to the slides and preservation of morphology of OS epithelial regions was achieved with 70% EtOH pre-chilled at −20°C.

### Number of sections on each slide

The influence of the number of tissue sections on the PALM slide was investigated using 1–6 sections per slide. It was noted that, more than 5 sections caused crowding of the tissue sections on the slide leading to overlapping. This resulted in loss of tissue for LMD and less RNA yield. Taking 1 to 2 sections per slide did not have an adverse effect on the RNA quality; but this was not cost effective. However, the cost effectiveness was maximised by taking 3 to 4 tissue sections on the PALM slide.

### Processing of the tissue sections for LMD

Pre LMD tissue section staining was performed with RNase free toluidine blue dye in combination with DEPC treated water (RNase free) for better identification of tissue morphology without compromising RNA integrity [16]. To maintain the integrity of the tissue RNA, LMD of the toluidine stained tissue sections was performed within 3 hours of the staining.

### Optimisation of the laser function for LMD

Adjustment of the laser settings at the beginning of the LMD is crucial for efficient LMD. This was achieved by positioning the slide on the robostage with 40X magnification and the stromal tissue furthest away from the epithelium, was brought into focus. A freehand drawing tool element was selected on the graphics toolbar, and an outline was drawn on the stromal side of the section. The laser speed was set to slow, following which the laser was activated by selecting the laser icon on the computer window screen. The UV energy, laser focus and laser speed were all adjusted and optimised until a thin white laser cut line was achieved. The laser adjustments also depended on the thickness of the tissue and the degree of hydration. In the current study, the laser settings for OS epithelial regions were as follows: UV energy 58-70 μJ, UV focus 75-80 μm, and UV speed 60-70 μm^2^/sec. Optimisation of the energy, focus and speed of the laser beam prior to LMD was found to be crucial for effective LMD. At high settings of the laser power (>75 μJ) singing or burning of the surrounding tissue was noted. In these circumstances, there was increasing possibility that laser might burn the adjacent epithelial cell layer during LMD, hence adversely influencing the molecular composition of the cells. When mild laser beam power was applied (<50 μJ) the tissue was not effectively cut, nor was successfully catapulted into the collection tube, thus requiring repeat laser. The areas of epithelium dissected for different samples ranged from approximately 80,000 μm^2^ to 300,000 μm^2^ depending on cellular density, which varied between epithelial regions. The minimal cut off of 80,000 μm^2^ was determined by optimising the LMD tissue area (71,284 μm^2^) required to obtain adequate RNA sample for successful semi-quantitative PCR analysis of Hypoxanthine Phosphoribosyltransferase 1 (HPRT1). In this study, no relationship was noted between the area of LMD tissue processed and the RNA concentration obtained, due to variable cellular density of each region (Table 1).

**Table 1:** **Details of samples processed for spotted microarray showing LMD area**, **corresponding concentration**, **of unamplified**, **amplified and labelled RNA samples with FOI values**

**ch1: source name**	**LMD area, μm**^**2**^	**RNA Conc pg/μl**	**Conc of amplified RNA ng/μl**	**Labelled extract ng/μl**	**FOI of Cy5 dye**
SC-CO398 Cornea	233,571	52.87	368.5	423.2	32
SC-CO399 Cornea	223,254	92.88	160.7	309	32
SC-CO418 Cornea	200,597	40.77	270.7	319.9	30.25
SC-CO398 Limbus	221,436	47.41	301.6	394.9	40
SC-CO399 Limbus	246,046	49.24	359.7	176.5	32.6
SC-CO404 Limbus	310,000	66.68	717.5	120.3	34.7
SC-CO418 Limbus	212,236	47.16	248.8	220	38
SC-CO398 LEC	86,251	35.95	232.3	127.2	35.2
SC-CO399 LEC	192,689	32.03	429.6	79.3	42
SC-CO404 LEC	279,404	80.59	581.2	249.2	41.3
SC-CO418 LEC	122,123	48.54	154.5	134.2	22
SC-CO399 Conj	214,212	24.54	274.1	151.1	41
SC-CO404 Conj	213,909	48.39	289.5	366.1	30
SC-CO418 Conj	151,862	62.3	330.4	254.6	41.27
SC-CO398 LEC Stroma	302,869	53.86	392	73.6	38
SC-CO399 LEC Stroma	392,887	46.03	542.8	109.4	46
SC-CO404 LEC Stroma	283,421	36.67	624.8	293.5	40.9
SC-CO418 LEC Stroma	320,055	26.31	324.1	551.9	33
**Ch2: source name Corneal + Conj epithelium**		**RNA conc μg/μl**	**2nd Round amplified RNA ng/μl**	**Labelled extract ng/μl**	**FOI of Cy3 dye**
SP-C0479 SP1		2.22 +8.98	912.9	641.9	31.6
SP-C0479 SP2			912.9	496.7	40

This table presents the details of the samples used for spotted oligonucleotide study, including LMD area with corresponding RNA concentration of the samples. Quality of the RNA was ascertained by nanodrop spectrophotometry. All the samples were efficiently processed as demonstrated by optimal range of FOI between 30 to 60 pmols/μl.

LEC had the highest density of cells and yielded a good concentration of RNA inspite of variations in number and sizes of LEC laser microdissected. However, the LEC stroma had sparse distribution of cells hence large areas from this region were laser microdissected to obtain sufficient concentrations of RNA.

### Optimisation of LMD tissue for Oligonucleotide microarray experiments

Four biological replicates from four pairs of eyes were processed for each OS epithelial region including the LEC, limbus, cornea, conjunctiva and LEC stroma. Table 1, shows unamplified, amplified RNA concentration values including the labelled probes of all the LMD samples processed in this microarray study. It also includes the concentration values of the reference probes (SP) and the concentrations of labelled extracts of samples and the Standard Probes with the Frequency of Incorporation (FOI) of the dyes in these samples. The labelled probes of the samples were matched to the Standard Probe with similar FOI to generate hybridised probes.

**Figure 4:**
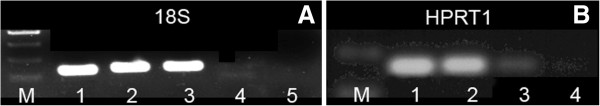
**Composite image of nanodrop spectrophotometry of amplified and labelled probes of spotted oligonucleotide microarrays.** Images **A** and **B** are nanodrop spectrophotometry of second round of amplification and labelled probe of a microarray sample (C0404 LEC) respectively. These graphs were used to determine the quality of the amplification and labelling of the sample for further processing. Image 4**A** of the amplified RNA demonstrates RNA concentration and 260/280 ratio which shows absence of protein contamination. The overall graph curve indicates good quality of amplified RNA. Image 4**B** of the labelled probe gives the concentration of the sample with frequency of incorporation of the dye in the sample.

The area of LMD tissue processed for spotted oligonucleotide microarray study ranged from 86,253 μm^2^ in LEC to 392,887 μm^2^ in LEC stroma (Table 1). RNA concentration of LEC samples measured with Agilent bioanalyser was found comparable to other regions in spite of small LMD area due to high cellular density in this region. In our experiments LMD samples of 80,000 to 300,000 μm^2^ yielded RNA concentrations of 22 to 92 pg/μl (Table 1). Samples of adequate concentration are crucial for microarray experiments. This was facilitated with use of Ambion® Message Amp™ II aRNA Amplification Kit which amplified as little as 100 pg of input RNA up to 1000 folds for Gene Chip® analysis as our samples fulfilled the optimal concentration required by this kit for further processing. According to manufacturer’s protocol we had optimised the starting RNA concentration of all the samples to 0.2 ng/10 μl for spotted oligonucleotide microarray study. The samples were again evaluated after each amplification round with quality control checks by NanoDrop ND-1000 UV–vis Spectrophotometer (Labtech International Ltd–UK), (Figure 4A) [17]. Samples with low 260/280 and 230/280 ratios (<1.8) were excluded from the study due to possible contamination with proteins and organic contaminants respectively. Unsatisfactorily amplified samples with low RNA concentrations and poor graph curves were also omitted from further processing [18]. The concentration of the labelled aRNA and FOI of the Cy5 and Cy3 dyes were measured using Nanodrop spectrophotometer. Labelled probes demonstrating optimal FOI between 30–60 incorporated dye molecules per 1000 nucleotides were included in the study (Table 1, Figure 4B).

### Validation of RNA viability with semi quantitative PCR

**Figure 5:**
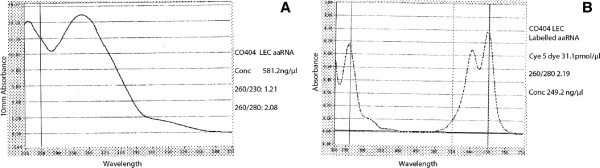
**Gel electrophoresis image showing expression of 18S and HPRT1 in LMD tissue.** The gel electrophoresis image shows strong expression of 18S in all the LMD samples (1, 2 and 3) irrespective of amount of LMD tissue dissected (Image 5**A**). Well number 4 and 5 is of the positive and negative control respectively, faint band is noted for the positive control. The gel image 5**B** shows strongly expressed HPRT1 bands in the LMD samples 1 and 2. Weak expression of HPRT1 was noted in sample 3, no bands were expressed in negative control sample (well 4).

Gel electrophoresis showed prominent expression of *18 s rRNA* in all the cDNA samples studied. The bands were not expressed in negative control samples. This result supports the absence of nonspecific PCR results and that sufficient RNA was obtained from LMD tissue for efficient and accurate amplification of the gene. However, *18 s rRNA* is an abundantly transcribed gene; hence it easily amplifies, the gel electrophoresis result had shown expression of intense bands with different areas of LMD samples (Figure 5A). *HPRT1* was more sensitive for accessing the RNA quality as it is a constitutively expressed housekeeping gene with low copy numbers (1–10 molecules/cell). Electrophoresis of *HPRT1* showed PCR amplification bands in all the samples at 159 Base Pair Sequence (BPS) of DNA ladder with absent expression in negative control sample (Figure 5B).

### Optimisation of LMD tissue for Gene 1.0 ST array

**Figure 6:**
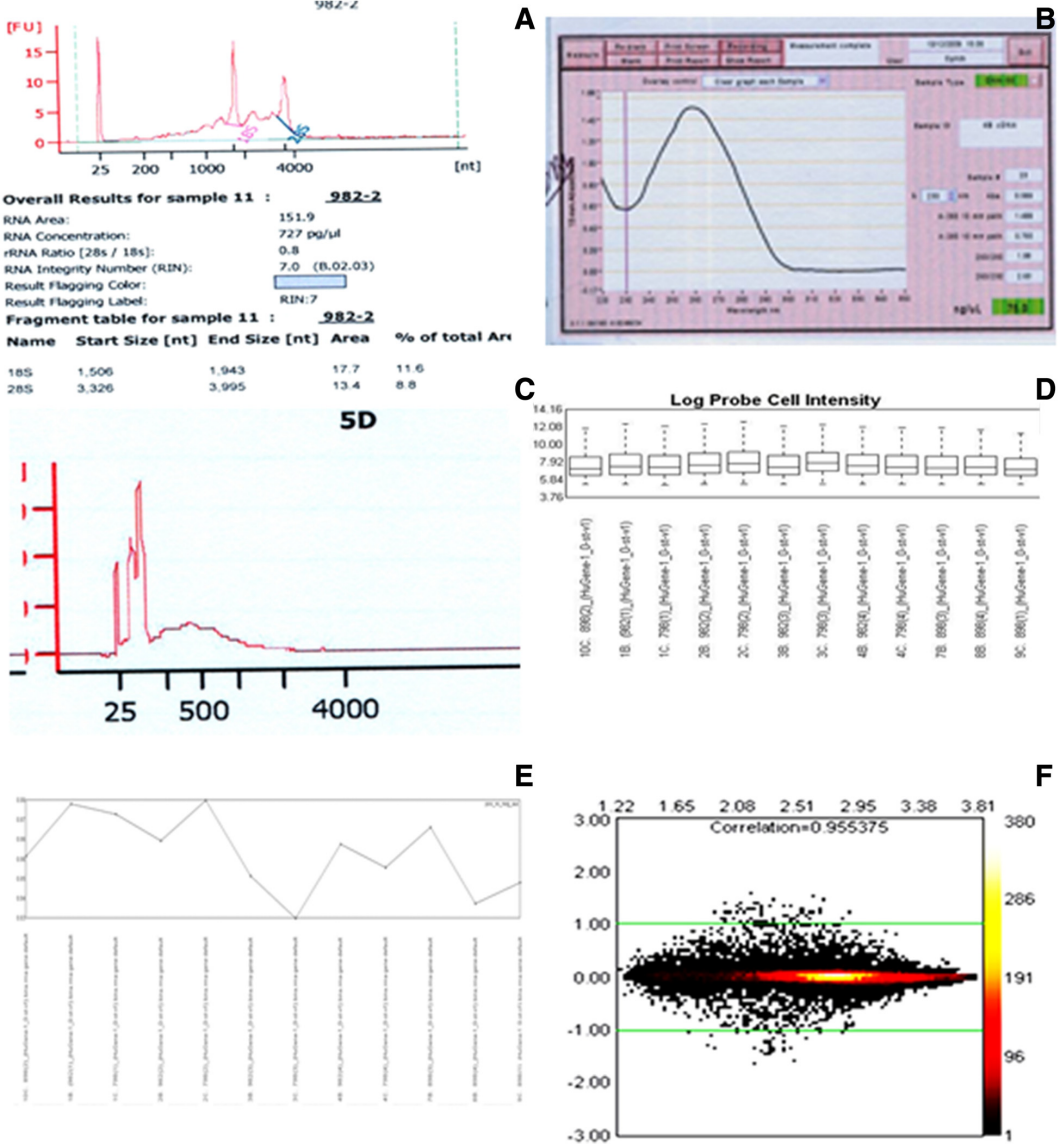
**Composite image of Quality Control metrics applied in Gene 1**.**0 ST array.** Image **A** is of an Agilent assay of a RNA sample showing two peaks 18S and 28S of ribosomal RNA. Image **B** is of the nanodrop spectrophotometer, the plot shows a good curve with 260/280 optical density ratio in the range of 1.8 to 2.2. Image **C** is of a nanodrop spectrophotometer plot of fragmented cDNA. The fragmentation efficiency of the fragmented sense strand cDNA was assessed with nanodrop. The fragmented cDNA peaks for the samples checked on Agilent Assay ranged between 40–70 NT (nucleotides); indicating efficient fragmentation of the samples. Image **D** is a box plot of probe cell intensities generated from the CEL file probe intensity values of all the 12 arrays before normalisation of the data. The expression intensity values are distributed around the median intensity values of the entire samples box plot. Image **E** is of a scatter graph plot of Pos_vs_neg_auc values across the samples. As seen in the figure all the samples were above the minimum criteria of 0.8. Image **F** shows MvA plot comparing two arrays 898(2) limbus and 798(1) cornea. The arrays show a good correlation of the expressed genes. The colour coding of the plot denotes the density of the signal probes represented by that data point. Y-axis represents the M values that display the differences in the log signals of the arrays and X-axis represents A values which is the average log signal. The green lines are the threshold lines for +/− two fold changes. The genes expressed around the baseline 0.00 are unchanged genes between the two arrays.

Gene 1.0 ST array experiments and real time PCR experiments for validation of endogenous control genes in the OS epithelial regions were performed with amplification of laser microdissected samples with NuGen WT-Ovation™ Pico RNA Amplification System. There was no correlation between post-mortem time and quality of RNA obtained however, because of the small sample size a firm conclusion on this cannot be made. The recommended starting concentration of the RNA samples used for Gene ST 1.0 arrays is 6 ng/5 μl [19]. As the recommended starting RNA concentration was much higher for Gene 1.0 ST arrays compared to the spotted oligonucleotide microarrays the area of LMD tissue processed for each sample in the Gene 1.0 ST array experiments was increased to approximately 100,0000 μm^2^ to 130,0000 μm^2^ (Table 2). On Agilent assay, the RNA samples exhibited both the 18S and 28S peaks distinctly and the RNA concentration of these samples ranged from 10.88 ng/12 μl to 25.8 ng/12 μl, with the RIN for these samples from 3.3 to 7.9 (Table 2, Figure 6A). The average RIN value for the samples was 5.1. As seen in Table 2, although sample number 9 had unrecordable RIN it was still included in the microarray experiments as it had good RNA concentration and quality. According to the manufacturer’s criteria, samples with good RNA concentration but low or absent RIN value were included in the study if these had amplified satisfactorily and fulfilled quality control metrics. However, samples with low RIN values that had failed to amplify satisfactorily were discarded. Only those samples shown in Table 2 that had fulfilled the quality control metrics (Additional file 1, Figure 6A, B, C, D, E and F) were included in the study.

**Table 2:** **LMD samples used in Gene ST 1**.**0 array experiments**

**Sr No**	**Samples ID**	**Tissue**	**LMD area μm**^**2**^	**RNA conc (ng/μl**)	**RIN**
1	C0798 (1)	Cornea	1237928	1.542	7.9
2	C0798(2)	Limbus	1058825	1.093	3.3
3	C0798(3)	LEC	1102794	0.902	6
4	C0798(4)	Conj	1287990	0.987	5.7
5	C0982(1)	Cornea	1062468	2.1577	6.2
6	C0982(2)	Limbus	1103061	1.8111	7
7	C0982(3)	LEC	1267265	1.3428	4.6
8	C0982(4)	Conj	1108369	1.162	6.6
9	C0898(1)	Cornea	1035312	1.0687	-
10	C0898(2)	Limbus	1229731	1.5995	7.8
11	C0898(3)	LEC	1374860	1.0795	4.7
12	C0898(4)	Conj	1060222	1.257	6.3

This table presents the details of the samples used for Gene 1.0 ST array study, including LMD area with corresponding RNA concentration and RIN values of the samples.

### Optimisation of LMD tissue for real time PCR

Five cadaveric eyes were processed for real time PCR experiments to validate the oligonucleotide microarray studies. The LMD area ranged from 85,831 μm^2^ to 138,868 μm^2^. The OS region samples were processed in triplicates to demonstrate replicable results.

## Discussion

Successful isolation of the tissue or cells by LMD involves appropriate collection or harvesting of the donor, with contamination free sectioning, processing and storage of the samples at −80°C to preserve the RNA quality prior to LMD.

Mechanical isolation of the limbal epithelium and the LEC without contamination from adjacent tissue is technically challenging, as the limbus is a narrow one mm transient zone between the cornea and conjunctiva and LEC is a deep seated structure arising from the under surface of limbus extending into the underlying limbal stroma. Similar observation regarding the limbus was previously made in a molecular study using LMD tissue [20]. LMD offers a viable and effective option to isolate cell populations of interest from heterogeneous tissue thus preserving the purity and integrity of the RNA samples for further molecular studies [4].

Pinzani *et al.* in 2006 had demonstrated that directly immersing the tissue in liquid nitrogen for cryopreservation disrupts the tissue morphology due to the formation of ice crystals leading to freeze fractures in the tissue. The authors have further suggested that controlled freezing of the tissue prevents the development of freeze fractures in the tissue and have demonstrated extraction of good quality RNA and DNA from the controlled frozen tissue [21].

The authors have further noted that tissue sections of 5-8 μm thickness are ideal for microscopic resolution due to monolayer cell thickness of these tissue sections. A similar protocol study on LMD tissues have reported effective RNA extraction of LMD tissue processed from 8 μm tissue cryosections [22].

In this study, cryosections of 6 μm thickness was noted to improve the histology quality of sections and visualisation of the LEC.

In a study on LMD tissue, Kerman *et al.,* had demonstrated a relationship between sectioning strategy and the RNA quality. They had observed that mounting 1–2 sections on the slide had minimal influence on the RNA quality; however, the RNA quality dropped when four or more sections were mounted per slide. The authors had noted that mounting more sections on a slide caused longer exposure of the sections to the room temperature while being processed, leading to uncontrolled RNase activity and hence RNA degradation during this period. Based on the abovementioned observation and our own experience, we had limited maximum of 4 sections on each PALM slide. This not only improved the cost effectiveness but also maintained the RNA quality of the processed tissue at the same time.

Furthermore, it was noted that post-staining handling of the slides for LMD for up to 3 hours did not significantly influence the quality of RNA. However, this processing time should not be exceeded; as such delays adversely affected the RNA quality [23]. Based on observations of this study all the toluidine stained tissue sections were laser microdissected within 3 hours of processing.

A study on LMD process has established better preservation of tissue morphology following fixation with 70% acetone at −20°C compared to 70% EtOH however, the RNA recovery was similar in both methods. For optimal results, the authors have suggested that tissue sectioning should be carried out at −20°C and have recommended use of membrane coated slides for excellent tissue capture during LMD [24]. Similarly, in our study, soon after the tissue sections were taken on the PALM slide the slides were retained in the cryostat chamber at −20°C until fixed in pre-chilled (−20°C) 70% v/v EtOH to prevent RNase activity. We had noted good preservation of morphology of OS epithelial regions following fixation in 70% EtOH during optimisation process.

A LMD study had demonstrated the effective staining of the tissue sections with toluidine blue, a nuclear dye resulting in preservation of RNA integrity and reliable amplification of cDNA with Taqman real-time PCR [25]. Similarly studies on the staining procedures for LMD sections, have suggested the use of RNase free aqueous staining solutions for preparation of the toluidine dye and washes to protect RNA integrity [26]. The authors of the latter study have recommended processing of the LMD tissue under sterile conditions and storage at −80°C in the lysis buffer until further use. During RNA extraction, they have advised DNase treatment of the samples to prevent genomic DNase contamination [22].

Comparison of RNA extraction of the LMD tissue with Qiagen RNeasy Micro kit (Valencia, CA) and Trizol RNA isolation reagent (Life Technologies, Invitrogen, Carlsbad, CA) have demonstrated efficient RNA extraction from LMD frozen tissue with Qiagen RNeasy Micro kit (Valencia, CA) [21].

In this study, dissection of variable amounts of epithelial tissues was unavoidable due to factors such as limited availability of LEC samples and sparse cellular density of LEC stroma. This limitation was dealt with by scrutinising wet unstained 6 μm thick serially cut cryosections involving 360° of the limbus, for LEC and including all the LEC sections for LMD to maximise the tissue availability. For LEC stroma samples large area of tissue was laser microdissected to obtain adequate RNA from this region.

### Potential pitfalls

During LMD due to the absence of cover slip over the tissue section visualisation of the morphology of the tissue becomes difficult therefore to facilitate identification of the morphology the tissue sections needs to be stained this can affect RNA viability.

LMD products in lysis buffer and RNA samples are unsuitable for long term storage.

Molecular study involving pure cell populations might not consider interaction between the cell population studied and surrounding structures such as supporting cells and the extracellular matrix. This may result in lack of information regarding cell to cell or cell to extracellular matrix signalling.

Certain downstream molecular procedures such as microarray experiments and proteomics may require large amount of samples, which may not be feasible to collect with LMD.

LMD needs to be performed in a limited period of time, as degradation of tissue samples may be an issue due to exposure during prolonged laser microdissection.

The amount of tissue sample needed is also determined by amount of target molecule per cell and number of replicates required.

### Trouble shooting

This section mainly presents the effect of optimisation of LMD technique with consideration to cryopreservation of the tissue, thickness of the cryosections taken on PALM slides, fixation technique and tissue staining (Table 3).

**Table 3:** Troubleshooting in Laser microdissection of OS regions

**Step**	**Problem**	**Reason**	**Solution**
Histological Staining			
	Contamination of samples	Contamination of contact surfaces such as cryostat blade, staining solutions and tissue slides with RNase	Use gloves and follow sterile method of sample preparation. Wipe down the work surfaces and instruments with Trigene, alcohol and also possibly with RNase zap sprays. Change cryostat blade after every use. Use fresh staining solutions which are prepared in RNase free solutions for tissue staining. Use sterile PALM slides and restrict the number of tissue sections per slide to 3–4. Don’t allow the tissue sections to thaw unnecessarily as it activates the RNases in the tissue.
	Unsatisfactory tissue staining resulting in difficulty in identification of tissue morphology in microscope.	Using too diluted staining solution. Incubation of tissue sections in staining solution inadequately. Thick tissue sections.	Use predetermined and optimised concentration of the staining solutions. Follow the recommended staining procedure. Increase incubation for staining.
Laser microdissection			
	Inadequate laser microdissection of tissue section	Thick tissue sections, tissue section placed near the margins of the slide resulting in tissue section or part of it not in the laser optical plane.	Ensure adequate dehydration of tissue section following staining procedure. This could be noted by checking for watermarks on the membrane of the PALM slide. Place tissue sections in the centre of the membrane oriented parallel with each other to prevent overlapping and folding of the sections.
	Failure or misdirection of cut tissue segment to catapult in the collection tube	Tissue section not dehydrated satisfactorily.	Prior to beginning of the LMD optimise the laser settings to facilitate adequate cutting without singing or burning of the surrounding tissue seen as black frill around the cut edges.
		Laser used is either out of focus or of inadequate power.	Small tissue segments are effectively catapulted in the collection tube
		Large area of tissue segment dissected	Adequate dehydration of the slide could be ensured by incubating the slide in warm air incubator for approximately 5 minutes or fixing the stained slide in the ethanol bath
		Tissue section not adequately dehydrated	Following LMD scan the slide surface under microscope to detect any misdirected pieces which could be redirected in collection tube by realigning the collection tube over the tissue segment and recatapultion of tissue segment with LPC laser function
		Inadequate laser power	Check the collection tube cap for number of tissue segments catapulted in comparison to actual LMD segments
RNA Extraction			
	Inadequate RNA quality and quantity	RLT buffer leaks from the collection tube	Pooling of the RNA samples from same biological replicate
		Contamination of RNA samples while processing	
		Variable amounts of RNA	

## Conclusions

LMD is an effective non-contact method of collection of tissue of interest from heterogeneous tissue samples for downstream molecular experiments. Optimisation of tissue collection, processing and LMD technique was found to be crucial to maximise the effectiveness of LMD process for generation of viable RNA samples of sufficient concentration and quality. We have demonstrated that picogram to nanogram amounts of laser microdissected RNA samples generated from *insitu* OS epithelial regions of human donor eyes can be successfully used for downstream molecular analysis such as microarrays and real time PCR.

## Competing interests

The authors declare that they have no competing interests.

## Authors’ contributions

BBK performed sample collection, microarray experiments and analysis of experimental data. HSD and DGP supervised the project and helped with planning of the experimental design. HSD and AH looked after the administration and the financial aspect of the project. BBK created draft of the manuscript which was critically revised edited and proof read by HSD and AH. DGP supervised laser microdissection and helped with trouble shooting of the experiments. All authors read and approved the final manuscript.

## Pre-publication history

The pre-publication history for this paper can be accessed here:

http://www.biomedcentral.com/1471-2415/13/62/prepub

## Supplementary Material

Additional file 1 Assessment of quality of cDNA samples processed for Gene 1.0 ST arrays and real time PCR. Click here for file
