# Supplementary material for: Optimised laser microdissection of the human ocular surface epithelial regions for microarray studies
Source: BMC Ophthalmol. 2013 Oct 26;13:62. doi: 10.1186/1471-2415-13-62 (PMC4015997; doi:10.1186/1471-2415-13-62)
Supplement: Additional file 1 — Assessment of quality of cDNA samples processed for Gene 1.0 ST arrays and real time PCR. [file 1471-2415-13-62-S1.docx]

Additional file 1

Assessment of quality of cDNA samples processed for Gene 1.0 ST arrays and real time PCR

The amplified cDNA samples were assessed for quality and quantity on nanodrop spectrophotometer (Figure 6 **B**) before proceeding for fragmentation and biotin labelling. The total concentration of the cDNA samples ranged between 3.272 to 5.705 μg. Poorly amplified samples shaded in grey were discarded and not included in further microarray experiments. Amplified cDNA samples between 3 to 5 μg were suitable for hybridisation on to Gene 1.0 ST array. Following conversion of cDNA samples from antisense to sense strand the samples were fragmented and labelled. Quality control of the fragmented samples was performed with Nanodrop spectrophotometer as shown in Figure 6 **C** of the main manuscript. Samples with cDNA peaks between 40 to 70 NT (nucleotide) indicated successful fragmentation; therefore, these samples were further hybridised.

**Quality control metrics of the microarray data**

The analysis of the quality control (QC) metrics, which included the hybridisation controls and the sample metrics of the data, is given below. Microarray analysis of the raw data was only performed when the data passed the QC criteria.

**Array Sample Metrics**

The sample metric parameters evaluated the quality of the samples used for microarrays following hybridisation to the array slides.

**Probe cell intensity**

The probe cell intensity plot generated box plot of probe cell intensities for each array. The distribution of intensities on each array was compared to the median probe intensity value for the group. This allowed identification of any outlier array(s) with divergent probe intensity distribution relative to other arrays in the study. In this study the distribution of the probe cell intensities across the arrays was uniform with the absence of any outlier samples as shown in Figure 6 **D** of the main manuscript.

**Pos**_**vs**_**neg**_**auc**

All the samples in this study had pos_vs_neg_auc value between 0.83 and 0.88 as seen in Figure 6 **E** of the main manuscript. None of the samples in this study had values less than 0.8, which represented the outlier samples. This demonstrated robustness of the data (Table 1).

**Table 1** Pos_vs_neg_auc values for samples in Gene 1.0 ST array

| Sample no | Sample ID | Pos_vs_neg_auc values |
| --- | --- | --- |
| 10C | 898(2) | 0.86 |
| 1B | 982(1) | 0.88 |
| 1C | 798(1) | 0.87 |
| 2B | 982(2) | 0.86 |
| 2C | 798(2) | 0.88 |
| 3B | 982(3) | 0.85 |
| 3C | 798(3) | 0.83 |
| 4B | 982(4) | 0.86 |
| 4C | 798(4) | 0.85 |
| 7B | 898(3) | 0.86 |
| 8B | 898(4) | 0.83 |
| 9C | 898(1) | 0.85 |

Samples with borderline or suboptimal RIN values have been highlighted in yellow.

**All probe set mean**

The all probe set mean values for the biological replicates in the same group should be consistent as compared to the samples in different groups, which may vary. All_probeset_mean values for the replicates of different samples in Gene 1.0 ST arrays ranged between 6.7 and 6.8 (Table 2)

**Table 2** All_Probeset_Mean values for samples in Gene 1.0 ST array

| Samples | Sample ID | All_Probeset_Mean |
| --- | --- | --- |
| 10C | 898(2) | 6.79 |
| 1B | 982(1) | 6.77 |
| 1C | 798(1) | 6.78 |
| 2B | 982(2) | 6.78 |
| 2C | 798(2) | 6.80 |
| 3B | 982(3) | 6.79 |
| 3C | 798(3) | 6.84 |
| 4B | 982(4) | 6.79 |
| 4C | 798(4) | 6.81 |
| 7B | 898(3) | 6.78 |
| 8B | 898(4) | 6.83 |
| 9C | 898(1) | 6.84 |

Samples with borderline or suboptimal RIN values have been highlighted in yellow.

**All probe set RLE mean**

The differences noted between the biological replicates of the OS epithelial region samples from the same donor were 0.1 or less, indicating the absence of any outliers in samples (Table 3).

**Table 3** All probe set RLE mean values of samples in Gene ST 1.0 arrays

| Sample no | Sample ID | All_Probeset_RLE_Mean |
| --- | --- | --- |
| 1B | 982(1) | 0.29 |
| 2B | 982(2) | 0.22 |
| 3B | 982(3) | 0.27 |
| 4B | 982(4) | 0.22 |
| 1C | 798(1) | 0.29 |
| 2C | 798(2) | 0.30 |
| 3C | 798(3) | 0.38 |
| 4C | 798(4) | 0.25 |
| 9C | 898(1) | 0.32 |
| 10C | 898(2) | 0.27 |
| 7B | 898(3) | 0.22 |
| 8B | 898(4) | 0.28 |

Samples with borderline or suboptimal RIN values have been highlighted in yellow.

**Hybridisation Controls Quality Metrics**

The 20x eukaryotic hybridisation controls at_3’ and 5’ end of the cDNA samples were used to monitor the hybridisation efficiency of the samples. Bio B 5_at represented assay sensitive complexity ratio of (1:100,000) was expressed in all the samples. According to the criteria for hybridisation controls Bio B 5_at and Bio B 3_at should be called present 70 percent of times in the samples and BioC, Bio D and CRE should be expressed in the following increasing order: BioC < BioD < CRE. The results showed that Bio B 5’ and 3’ was consistently expressed in all the samples at similar levels. The signal values BioC, BioD, CRE for the spike controls at 5’ and at 3’ ends were found to increase in the order of BioC < BioD < CRE (Tables 4 and 5).

**Table 4** Expression of hybridisation controls in the OS epithelial regions at 5’ end of the cDNA samples

| Sample no | Sample ID | BioB-5_at | BioC-5_at | BioDn-5_at | Crex-5_at |
| --- | --- | --- | --- | --- | --- |
| 10C | 898(2) | 10.622309 | 11.655915 | 12.250127 | 13.814619 |
| 1B | 982(1) | 10.477743 | 11.480821 | 12.082915 | 13.716012 |
| 1C | 798(1) | 10.325689 | 11.340178 | 11.928886 | 13.646859 |
| 2B | 982(2) | 10.264658 | 11.310485 | 11.913496 | 13.571549 |
| 2C | 798(2) | 10.241383 | 11.259228 | 11.938232 | 13.58019 |
| 3B | 982(3) | 10.763971 | 11.786589 | 12.450968 | 13.887021 |
| 3C | 798(3) | 10.201749 | 11.315104 | 11.923002 | 13.511822 |
| 4B | 982(4) | 10.539779 | 11.584466 | 12.236828 | 13.768787 |
| 4C | 798(4) | 10.551792 | 11.529917 | 12.140465 | 13.687775 |
| 7B | 898(3) | 10.607733 | 11.580786 | 12.285089 | 13.814423 |
| 8B | 898(4) | 10.678362 | 11.673722 | 12.324007 | 13.690212 |
| 9C | 898(1) | 10.68335 | 11.606025 | 12.259851 | 13.818562 |

Samples with borderline or suboptimal RIN values have been highlighted in yellow.

**Table 5** Hybridisation controls expressed in OS epithelial regions at 3’ end of the cDNA samples

| Sample no | Sample ID | BioB-3_at | BioC-3_at | BioDn-3_at | Crex-3_at |
| --- | --- | --- | --- | --- | --- |
| 10C | 898(2) | 11.008632 | 10.815928 | 13.557007 | 14.066576 |
| 1B | 982(1) | 10.830392 | 10.58568 | 13.465986 | 14.010655 |
| 1C | 798(1) | 10.749696 | 10.457777 | 13.485157 | 14.010273 |
| 2B | 982(2) | 10.659513 | 10.461383 | 13.388121 | 13.886829 |
| 2C | 798(2) | 10.583462 | 10.394482 | 13.409548 | 14.046679 |
| 3B | 982(3) | 11.22631 | 10.922373 | 13.634194 | 14.090215 |
| 3C | 798(3) | 10.698716 | 10.355176 | 13.419991 | 13.830913 |
| 4B | 982(4) | 11.052122 | 10.769809 | 13.590767 | 14.144723 |
| 4C | 798(4) | 10.931898 | 10.639848 | 13.47979 | 13.977884 |
| 7B | 898(3) | 10.942164 | 10.807373 | 13.557606 | 14.07262 |
| 8B | 898(4) | 11.06184 | 10.833438 | 13.490187 | 14.007899 |
| 9C | 898(1) | 11.128157 | 10.807057 | 13.562658 | 14.122297 |

Samples with borderline or suboptimal RIN values have been highlighted in yellow.

**MVA plot view**

MVA plot involved comparison between two CHP files for the arrays. In this study, 66 MvA plots were generated for combinations of 12 arrays. The Pearson correlation for the array combinations r^2^ ranged from 0.91 to 0.96. The arrays showed good concordance values as seen in Figure 6 **F** of the main manuscript.
